# Histological Alterations in Placentas of Pregnant Women with SARS-CoV-2 Infection: A Single-Center Case Series

**DOI:** 10.3390/pathogens12101197

**Published:** 2023-09-26

**Authors:** Jesus Enrique Patiño Escarcina, Ana Keila Carvalho Vieira da Silva, Malú Oliveira de Araújo Medeiros, Stephanie Santos Santana Fernandes, Luiza Andrade Agareno, Louise Andrade Garboggini, Marcela de Sá Gouveia, Vanessa Campos Duarte, Diogo Lago Morbeck, Lícia Maria Oliveira Moreira

**Affiliations:** 1 José Silveira Foundation, Center for Research, Learning and Innovation, Salvador 40210-320, BA, Brazil; anakeila.enf@gmail.com (A.K.C.V.d.S.); luizaagareno@gmail.com (L.A.A.); diogolm@ufba.br (D.L.M.); liciamom@gmail.com (L.M.O.M.); 2 Collective Health Institute, Universidade Federal da Bahia, Salvador 40110-040, BA, Brazil; 3 Bahiana School of Medicine and Public Health, Salvador 40290-000, BA, Brazil; malumedeiros19.1@bahiana.edu.br; 4 Faculty of Medicine, UniFTC University Center, Salvador 41720-200, BA, Brazil; stephanie.fernandes@ftc.edu.br; 5 Climério de Oliveira Maternity, Salvador 40055-150, BA, Brazil; lou.garbo@hotmail.com (L.A.G.); marcelagouveia@gmail.com (M.d.S.G.); vanessacduarte@hotmail.com (V.C.D.)

**Keywords:** SARS-CoV-2, placenta, pregnant, histopathology

## Abstract

This study aimed to investigate the histopathological changes associated with SARS-CoV-2 infection in placentas. A case series of anatomopathological analysis was conducted on the placentas of pregnant women with SARS-CoV-2 who delivered between March and December 2020 at Santo Amaro Hospital (HSA) in Salvador, Brazil. Out of the 29 placentas examined, the median weight was 423.0 (IQR: 385.0–521.0) g. Among them, 58.3% (*n* = 14) had inadequate weight relative to the newborn’s weight. The histopathological findings revealed that 86.2% (*n* = 25) of the placentas had poorly defined lobes, and the fetal and maternal surface color was normal in 89.7% (*n* = 26) and 93.1% (*n* = 27), respectively. Additionally, 51.7% (*n* = 15) of the umbilical cords displayed hypercoiling. The most frequent microscopic finding was infarction, present in 35.3% (*n* = 6) of the cases, followed by 11.8% (*n* = 2) for each of chorioamnionitis, chronic villitis, focal perivillositis, and laminar necrosis. Analysis of the umbilical cords identified 23.5% (*n* = 4) cases of intervillous thrombosis, while amnion analysis showed 13.8% (*n* = 4) cases of squamous metaplasia. Extraplacental membrane examination revealed fibrin deposition in 93.1% (*n* = 27) of the cases, necrosis in 62.0% (*n* = 18), calcifications in 51.7% (*n* = 15), cysts in 37.9% (*n* = 11), neutrophilic exudate in 17.2% (*n* = 5), thrombosis in 13.7% (*n* = 4), and delayed placental maturation in 6.9% (*n* = 2). All analyzed placentas exhibited histopathological changes, primarily vascular and inflammatory, which indicate SARS-CoV-2 infection in term pregnancies. These alterations could be associated with impaired placental function, fetal growth restriction, preeclampsia, and prematurity. However, further prospective studies are required to validate the type, prevalence, and prognosis of each of these changes.

## 1. Introduction

The severe acute respiratory syndrome coronavirus type 2 (SARS-CoV-2), responsible for the lung disease called COVID-19, was first identified in December 2019 in China [1]. Since its discovery, SARS-CoV-2 has rapidly spread worldwide, leading to a global pandemic state declared by the World Health Organization (WHO) in March 2020 [2]. This coronavirus is associated with fatal clinical conditions, having caused more than 6.5 million deaths and 600 million confirmed cases worldwide as of September 2022 [2], and has caused severe economic and social damage globally. Pregnant and postpartum women have been disproportionately affected, facing an increased risk of severe illness and death [3].

In the case of pregnant women, it is suspected that physiological changes, such as endocrinological adaptations [4], reduced respiratory volumes, edema of the respiratory tract mucosa [5,6], and immune alterations [7], condition their response to infections, especially viruses [8]. SARS-CoV-2 is known to infect nasal and oral mucosal cells using the angiotensin-converting enzyme receptor 2 (ACE2) and transmembrane serine protease 2 (TMPRSS2) [9], initiating viral replication, release, and an inflammatory process [10].

Some cases of maternal SARS-CoV-2 infection have been associated with placental changes, such as atheromas in the decidua vessels, poor blood perfusion, placental vasculopathy, placental infarction foci, chorioangioma, and inflammatory infiltrates with edema in the placental villi [11,12]. These abnormalities could be linked to the wide expression of ACE2 and TMPRSS2, the primary mediators of SARS-CoV-2 entry [9], and in cells of the female genital tract and the fetal-placental unit, including syncytiotrophoblasts, cytotrophoblasts, endothelial cells, and vascular smooth muscles of the primary and secondary villi [13]. The pathophysiology of SARS-CoV-2 infection may also negatively affect ACE2 gene expression, reducing the conversion of Angiotensin II to Angiotensin I–VII. Consequently, the histological characteristics of the placenta may increase its susceptibility to the new coronavirus during maternal infection, leading to an inflammatory reaction with vasoconstrictor, proliferative, and angiogenic effects. These phenomena could functionally compromise the placenta’s development, resulting in unfavorable repercussions during pregnancy, such as preeclampsia, premature birth, and an increased incidence of stillbirths [14,15]. Moreover, poor maternal vascular perfusion may be associated with more significant risks of impaired placental function and fetal growth restriction [16]. It is essential to recognize the placenta as a vital organ for maternal–fetal communication, the primary barrier against pathogens between mother and fetus, and which is responsible for maintaining and balancing endocrine-immunological factors for normal fetal development [17].

While some authors believe that neonatal repercussions associated with COVID-19 may be related to placental dysfunction [18], the available evidence remains unclear, with isolated cases recently reported in the literature [19].

Therefore, this study aims to describe the most frequent placental histopathological changes in parturients infected with SARS-CoV-2 treated at a maternity hospital in Salvador, Brazil. Understanding these changes can provide valuable insights into the impact of SARS-CoV-2 on placental health and contribute to improved maternal and fetal care during the COVID-19 pandemic.

## 2. Materials and Methods

A case series study was conducted to analyze the histopathology of placentas from pregnant women with COVID-19 who were treated at Santo Amaro Hospital (HSA), a private maternity managed by the José Silveira Foundation (FJS), a non-profit philanthropic institution with a long-standing presence in Salvador, Brazil. The study spanned cases between March and December 2020. Despite the ongoing COVID-19 pandemic, HSA continued its operations without interruption, implementing biosecurity measures and following recommendations from the Brazilian Ministry of Health and the World Health Organization.

At HSA, every pregnant woman displaying respiratory symptoms upon admission for either spontaneous labor initiation or scheduled cesarean section underwent SARS-CoV-2 testing through the real-time polymerase chain reaction (RT-PCR) assay (Xpert^®^ Xpress SARS-CoV-2; Cepheid Europe SAS, Maurens-Scopont, France). Some of the placentas were subjected to histopathological analysis based on specialist requests due to visible macroscopic abnormalities or the clinical characteristics of the pregnant women. The collected samples were carefully labeled and sent to the Pathology Department at HSA. Unaware of the patients’ details, two specialist physicians performed the histopathological analysis after obtaining consent from the mothers. The study included all available placental tissue samples with histological analysis collected during the data collection period.

For the histopathological analysis, placental tissue samples were fixed in 10% buffered formalin for approximately 24 to 48 h to ensure proper fixation before microscopic examination. The analysis covered macroscopic and microscopic descriptions of the placentas. Macroscopic characteristics were assessed, including weight, dimensions, fetal surface staining, transparency of extraplacental membranes, lobe formation, maternal surface staining, and cut surface aspects. Additionally, characteristics of the umbilical cord, such as the insertion location, length, number of vessels, maximum diameter, and number of turns, were examined along with features of the extraplacental membranes, such as the site of membrane rupture, integrity, color, and consistency. The microscopic examination involved analysis of the chorionic villi’s morphological characteristics according to gestational age. Inflammatory aspects of the samples were characterized, quantified, and described in terms of topography and the type of inflammatory infiltrate. The study also investigated factors related to poor maternal and fetal vascular perfusion.

It is essential to note that the study could not assess the presence of SARS-CoV-2 genetic material using the RT-PCR test due to technological and material constraints.

## 3. Results

### 3.1. Macroscopic Characteristics

During the study period, 29 placentas from pregnant women with COVID-19 underwent histopathological analysis. Among these placentas, five were associated with preterm births, and, notably, three of them were from a set of triplets, all of whom had a low birth weight. No cases of placental abruption were reported. The median placental weight was 423.0 g (interquartile range: 385.0–521.0 g). A total of 58.3% (*n* = 14) of all placentas had a weight lower than adequate for the newborn’s weight, 37.5% (*n* = 9) had a higher weight, and 4.2% (*n* = 1) had an adequate weight. In 92.6% (*n* = 25) of the cases, the placental histological characteristics were compatible with the gestational age.

Macroscopic features were considered normal in all cases. For most samples (89.7%; *n* = 26), the umbilical cord had a paracentral insertion, 6.9% (*n* = 2) had a central insertion, and 3.4% (*n* = 1) had a paramarginal insertion. The median cord length was 44.0 cm (interquartile range: 37.0–47.0 cm), and the median maximum umbilical cord diameter was 1.5 cm (1.3–1.8 cm). In 51.7% (*n* = 15) of the cases, hypercoiled cords (≥15 turns) were observed, while 48.3% (*n* = 14) had normal turns. The umbilical cord color was normal in 93.1% (*n* = 27) of the cases and greenish-brown in 6.9% (*n* = 2). All placentas had normal umbilical cord vessels.

All specimens preserved the extraplacental membranes and the umbilical cord’s transparency. In most analyzed placentas (89.7%, *n* = 26), the fetal surface color was violet, which is considered normal; 10.3% (*n* = 3) showed brown or greenish-brown coloration. The maternal surface exhibited normal color in 93.1% (*n* = 27) of the cases, while the remaining 6.9% (*n* = 2) had dark red coloration. Transparency was preserved in 100% of the studied samples. Trabeculation was observed in 82.8% (*n* = 24) of the placentas, with 13.8% (*n* = 4) being well-trabeculated and only 3.4% (*n* = 1) showing mild trabeculation. Most placentas had poorly delimited lobes (86.2%; *n* = 25), and 13.8% (*n* = 4) had well-delimited lobes. The appearance of the cut surface was spongy in all cases. Detailed characteristics are presented in Table 1.

### 3.2. Microscopic Findings

The histopathological analysis of the placentas revealed predominant vascular and inflammatory pathological changes. The most frequent findings were placental infarction and chorioamnionitis, each accounting for 20.6% (*n* = 6) of cases, followed by villitis and perivillositis at 13.8% (*n* = 4), intervillositis at 10.3% (*n* = 3), and tissue laminar necrosis at 6.9% (*n* = 2).

Regarding the microscopic examination of the cord, 96.6% (*n* = 28) of the parturients had chorionic villi compatible with the gestational age, with only one case (3.4%) being incompatible. The amniotic epithelium was cylindrical or cuboidal in 96.4% (*n* = 27) of patients, and only one case (3.4%) exhibited cuboidal epithelium without atypia, along with foci of squamous metaplasia. Squamous metaplasia and neutrophilic exudate were identified in equivalent proportions in 13.8% (*n* = 4) of the cases, while neutrophilic infiltrate was observed in 3.4% (*n* = 1).

**Figure 1:**
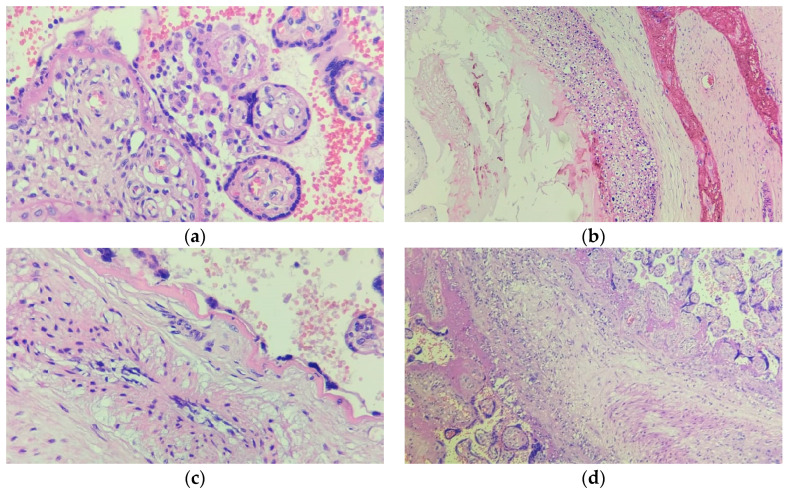
Key histopathological findings in the placental tissue of pregnant women with SARS-CoV-2 infection. (**a**) Intervillositis; (**b**) vasculitis accompanied by thrombus formation; (**c**) chorioamnionitis; (**d**) villitis.

In the extraplacental membranes, fibrin deposition was the most predominant finding (93.1%; *n* = 27), followed by necrosis (62.0%; *n* = 18), calcifications (51.7%; *n* = 15), cysts (37.9%; *n* = 11), presence of leukocytes (31.0%; *n* = 9), neutrophilic exudate (17.2%; *n* = 5), thrombosis (13.7%; *n* = 4), delayed placental maturation (6.9%; *n* = 2), and neutrophilic infiltrate (3.4%; *n* = 1). Table 2 provides a comprehensive overview of the specific characteristics, while Figure 1 highlights some key histopathological observations in the placental tissue.

## 4. Discussion

During the COVID-19 pandemic, pregnant and postpartum women experienced excessive mortality rates and lower admission rates to intensive care units than the general population [20]. This study aimed to evaluate the effects of SARS-CoV-2 on placental histology. Previous research reported maternal complications in women infected with SARS-CoV-2, such as oligohydramnios, polyhydramnios, and premature rupture of membranes, alongside placental abnormalities like placental abruption, placenta previa, and abnormal cord insertion [20]. However, detailed descriptions of placental abnormalities are limited. In this context, the study described the main histopathological findings in the placentas of SARS-CoV-2-infected women.

SARS-CoV-2, like other viruses [21], can invade placentas in infected pregnant women [22,23]. While some macroscopic placental abnormalities and inflammatory lesions have been linked to SARS-CoV-2 infection, there is no conclusive evidence of a typical COVID-19-specific pattern of placental pathology [24,25]. Notably, a considerable proportion of placentas in our study had less than adequate weight than the newborn’s weight, indicating the potential impact of acute COVID-19, which might affect fetal weight considering the placenta’s role in fetal development [26].

In our study, placental maturation was not impaired, as the histological characteristics were compatible with gestational age. However, some previous studies have indicated maturation defects, chorangiosis, and chronic deciduitis [27]. These discrepancies might be due to the short duration of placental affection in the cases of acute SARS-CoV-2 infection in our sample, which might not significantly impact placental maturation or fetal well-being [28].

Macroscopically, the placental morphology did not seem to be affected by acute SARS-CoV-2 infection as it was detected near the time of delivery. Normal color, transparency, insertion, and characteristics of umbilical cord vessels, placental surfaces, and extraplacental membranes were observed. However, over half of the placentas presented hypercoiled cords, poorly delimited lobes, and moderate trabeculation, which have been associated with chorioamnionitis and an increased risk of intrauterine growth restriction and severe neonatal outcomes, probably not associated with SARS-CoV-2 infection [29]. Limited evidence exists regarding macroscopic placental abnormalities associated with SARS-CoV-2 infection, with conflicting reports on the matter [30,31].

In the literature, placentitis has been frequently associated with SARS-CoV-2 infection [24]. Our microscopic analysis revealed cases of intervillous thrombosis, villitis, perivillositis, chorioamnionitis, and tissue necrosis, indicating that SARS-CoV-2 impacts placental vascularization and triggers an inflammatory response in the tissue. However, the association with maternal or fetal vascular changes remains unclear, as some studies reported similar odds of vascular changes between SARS-CoV-2 infected and healthy individuals [32]. In contrast, others reported increased arteriopathies, hypercapillarization, or villous tree immaturity [33]. In a few cases, these changes were associated with chronic histiocytic intervillositis, membrane hemorrhage, poor placental vascular perfusion, and an increase in perivillous fibrin deposition on the maternal side and occasionally severe outcomes such as fetal death [25]. Other findings previously reported but not identified in our analysis included increased meconium-laden macrophages in the amnion membrane, myometrial fibers attached to the basal layer, and microscopic accrete [24].

In our study, the analysis of extraplacental membrane samples revealed the predominant presence of fibrin deposition, necrosis, calcifications, cysts, and leukocytes. Several studies have reported similar findings associated with SARS-CoV-2 infection [24,25]. These findings indicate a placental inflammatory process related to viral invasion, potentially increasing the likelihood of severe maternal and fetal outcomes [33,34]. However, further research is required to establish a direct association with disease severity or progression, particularly concerning transplacental transmission and considering gestational age at the time of infection [35].

Our study encountered some limitations that should be acknowledged. Firstly, we were unable to conduct analyses of SARS-CoV-2 genetic material in the placenta tissue. However, similar investigations in syncytiotrophoblasts from other studies [36] have demonstrated elevated viral loads in the placenta compared to amniotic fluid or maternal and neonatal blood, lending support to the potential for vertical transmission [22]. Potential transmission routes may include transplacental or intrapartum transmission through contact with cervical and vaginal secretions, breastfeeding, or direct infection of fetal blood cells [37]. Neonatal infection rates have been reported to be around 3%, primarily associated with severe maternal COVID-19 requiring hospitalization in the intensive care unit (ICU) or resulting in maternal death [38,39]. In cases where neonatal infection was confirmed, intrauterine fetal distress, abortions, stillbirths, and preterm births were recorded [20,40]. Secondly, incorporation of a non-COVID-19 control group of placentas for comparison was unattainable due to the limited availability of data concerning other placental samples and maternal health conditions. Prior to the COVID-19 pandemic, anatomopathological analysis at HSA was typically conducted immediately after clinical decisions were made based on macroscopic observations during delivery, alongside patient consent. Conversely, amid the COVID-19 pandemic, the same criteria were followed, but SARS-CoV-2 detection was exclusively carried out for symptomatic pregnant individuals, which has the potential to inadvertently categorize asymptomatic or mildly symptomatic SARS-CoV-2 cases as controls. Lastly, along similar lines, our study exclusively incorporated confirmed COVID-19 cases through genetic material detection in symptomatic pregnant women. This approach omitted an undisclosed count of asymptomatic or mildly symptomatic COVID-19 cases where placental analyses were not conducted, thereby restricting the scope of our study sample.

Despite the acknowledged limitations of our study, our findings uncover significant histological alterations that offer valuable insights for both clinical practice and future research in this field. These findings are in line with previous research, underscoring the importance of vigilant monitoring for pregnant individuals with COVID-19 due to potential implications for both maternal and fetal well-being. Further investigations are imperative to unravel the intricate associations between histological findings and SARS-CoV-2 infection, considering various disease characteristics, the timing of infection, and potential neonatal repercussions. Additionally, it is essential that future studies delve into the long-term consequences of these histological changes and their possible impact on vertical transmission and neonatal outcomes. Armed with this knowledge, clinicians can develop more precise management strategies and interventions tailored to pregnant women affected by SARS-CoV-2 infection, ultimately enhancing the care and outcomes for both mother and child. The significance of these alterations underscores the ongoing need for careful monitoring of pregnant individuals with COVID-19, given their potential implications for maternal and fetal health.

## 5. Conclusions

The histopathological analysis of the placentas revealed changes that may be linked to acute infection by the novel coronavirus. This study describes the primary histological changes observed in the placentas of pregnant women with SARS-CoV-2, which align with similar findings in diverse populations, emphasizing the potential of SARS-CoV-2 to induce histological alterations, particularly with an inflammatory and vascular nature.

## Figures and Tables

**Table 1:** Macroscopic placental characteristics of pregnant women with COVID-19.

Placental Characteristics		N = 29
Weight (g)		423.0 (385.0–521.0)
Placental weight in relation to newborn weight	Less than adequate	14 (58.3%)
	Higher than adequate	9 (37.5%)
	Adequate	1 (4.2%)
Width (cm)		18.0 (16.5–20.5)
Length (cm)		16.0 (15.0–18.0)
Thickness (cm)		2.7 (2.5–3.0)
Fetal surface staining	Violet	26 (89.7%)
	Brown	2 (6.9%)
	Greenish-brown	1 (3.4%)
Trabeculation	Well-trabeculated	4 (13.8%)
	Moderate	24 (82.8%)
	Discreet	1 (3.4%)
Placental lobes	Poorly delimited	25 (86.2%)
	Well-delimited	4 (13.8%)
Maternal surface staining	Normal	27 (93.1%)
	Dark red	2 (6.9%)
Insertion of the umbilical cord	Central	2 (6.9%)
	Paracentral	26 (89.7%)
	Paramarginal	1 (3.4%)
Umbilical cord length (cm)		44.0 (37.0–47.0)
Greenish-brown umbilical cord staining		2 (6.9%)
Maximum diameter of the umbilical cord (cm)		1.5 (1.3–1.8)
Cord with more than 15 turns		15 (51.7%)

**Table 2:** Microscopic placental characteristics of pregnant women with COVID-19.

Placental Characteristics		N = 29
Microscopic examination of the umbilical cord	Neutrophilic exudate	4 (13.8%)
	Amnion with squamous metaplasia	4 (13.8%)
	Neutrophilic infiltrate	3 (10.3%)
	Cuboidal without atypia, foci of squamous metaplasia	1 (3.6%)
	Amnion with neutrophilic infiltrate	1 (3.4%)
Extraplacental membranes	Fibrin deposition	27 (93.1%)
	Presence of necrosis	18 (62.0%)
	Calcifications	15 (51.7%)
	Cysts	11 (37.9%)
	Presence of leukocytes	9 (31.0%)
	Presence of neutrophilic exudate	5 (17.2%)
	Thrombosis	4 (13.8%)
	Delayed placental maturation	2 (6.9%)
	Presence of neutrophilic infiltrate	1 (3.4%)
Microscopic examination of the placenta	Placental histology compatible with gestational age	27 (93.1%)
	Chorionic villi compatible with gestational age	28 (96.6%)
	Placental infarction	6 (20.6%)
	Intervillous thrombosis	6 (20.6%)
	Chorioamnionitis	6 (20.6%)
	Villitis	4 (13.8%)
	Perivillositis	4 (13.8%)
	Intervillositis	3 (10.3%)
	Tissue laminar necrosis	2 (6.9%)

## Data Availability

The authors confirm that the data supporting the results and findings of this study are available within the article. The datasets generated and analyzed during the current study are not publicly available due to privacy but are available from the corresponding author upon reasonable request.
